# Single-pulse enhanced coherent diffraction imaging of bacteria with an X-ray free-electron laser

**DOI:** 10.1038/srep34008

**Published:** 2016-09-23

**Authors:** Jiadong Fan, Zhibin Sun, Yaling Wang, Jaehyun Park, Sunam Kim, Marcus Gallagher-Jones, Yoonhee Kim, Changyong Song, Shengkun Yao, Jian Zhang, Jianhua Zhang, Xiulan Duan, Kensuke Tono, Makina Yabashi, Tetsuya Ishikawa, Chunhai Fan, Yuliang Zhao, Zhifang Chai, Xueyun Gao, Thomas Earnest, Huaidong Jiang

**Affiliations:** 1 State Key Laboratory of Crystal Materials, Shandong University, Jinan 250100, China; 2 Chinese Academy of Sciences Key Laboratory for Biomedical Effects of Nanomaterials and Nanosafety, Institute of High Energy Physics, Chinese Academy of Sciences, Beijing 100049, China; 3 RIKEN SPring-8 Center, Kouto 1-1-1, Sayo, Hyogo 679-5148, Japan; 4 Department of Physics and Astronomy and California NanoSystems Institute, University of California Los Angeles, California 90095, USA; 5 School of Materials Science and Engineering, Gwangju Institute of Science and Technology, Gwangju 61005, Korea; 6 Department of Physics, POSTECH, Pohang 790-784, Korea; 7 Japan Synchrotron Radiation Research Institute (JASRI), 1-1-1 Kouto, Sayo, Hyogo 679-5198, Japan; 8 Shanghai Synchrotron Radiation Facility, Shanghai Institute of Applied Physics, Chinese Academy of Sciences, Shanghai 201800, China; 9 iHuman Institute, Shanghai Tech University, Shanghai 201210, China; 10 School of Physical Science and Technology, Shanghai Tech University, Shanghai 201210, China

## Abstract

High-resolution imaging offers one of the most promising approaches for exploring and understanding the structure and function of biomaterials and biological systems. X-ray free-electron lasers (XFELs) combined with coherent diffraction imaging can theoretically provide high-resolution spatial information regarding biological materials using a single XFEL pulse. Currently, the application of this method suffers from the low scattering cross-section of biomaterials and X-ray damage to the sample. However, XFELs can provide pulses of such short duration that the data can be collected using the “diffract and destroy” approach before the effects of radiation damage on the data become significant. These experiments combine the use of enhanced coherent diffraction imaging with single-shot XFEL radiation to investigate the cellular architecture of *Staphylococcus aureus* with and without labeling by gold (Au) nanoclusters. The resolution of the images reconstructed from these diffraction patterns were twice as high or more for gold-labeled samples, demonstrating that this enhancement method provides a promising approach for the high-resolution imaging of biomaterials and biological systems.

Understanding the spatial and temporal regulation of biological assemblies within the cell remains a fundamental challenge of modern biology. Whole-cell, high-resolution imaging is one of the primary goals of microscopy. The diffraction limits of visible light^1^ sets the resolution of conventional optical microscopy to approximately 200 nm, although super-resolution optical approaches enable the capture of images at higher resolution than the wavelength of visible light in certain cases^2^. However, obtaining this higher resolution requires the use of fluorescent labels; therefore, only the positions of the labels are localized, not the full cell contents. Transmission electron microscopy can also achieve high-resolution images^3^,^4^ but is limited by sample thickness to less than that of most cells^5^, unless sectioning approaches are used. X-rays are an ideal source for the high-resolution imaging of thick specimens because of their short wavelength and deep penetration^6^. Unfortunately, the spatial resolution of conventional X-ray microscopy is limited by X-ray focusing devices^6^, such as zone plates. Coherent X-ray diffraction imaging (CDI) enables the high-resolution imaging of thick samples. This technique is based on the principle that the oversampled coherent diffraction patterns obtained from samples are recorded and then directly computationally phased to obtain a reconstructed real-space image using iterative algorithms^7^. Since the first demonstration by Miao *et al*.^8^, CDI has been applied to imaging a wide range of 2D and 3D materials at nanoscale resolution^9^,^10^,^11^,^12^,^13^,^14^,^15^,^16^. Due to the low scattering power of biological samples and the problems associated with radiation damage, resolution of only a few tens of nanometers has been achieved for biomaterials^17^. Numerical simulations indicate that X-ray free-electron lasers (XFELs) can overcome the problems associated with radiation damage; theoretically, atomic resolution is achievable using ultra-bright and ultra-fast single pulses^18^,^19^,^20^. Thus, a combination of this methodology with XFELs might provide a way to study complex biological systems in structural biology^21^. Recently, these techniques have been applied to noncrystalline biomaterials, such as a mimivirus^22^, an RNAi microsponge^23^ and live cells^24^, using the “diffraction before destruction” method^25^.

Due to the low scattering ability of biomaterials, the achievable resolution is less than that required to study cells and their internal architecture at the nanometer scale even using an ultra-bright XFEL light source. Several simulation results based on adding heavy-atom templates as reference objects have shown that the resolution can be increased by combining this technique with the coherent diffraction imaging method. In this study, we demonstrated a method that can enhance resolution by labeling cells with heavy atoms. A comparison of the results from reconstructions of labeled and unlabeled (control) cells shows that the achievable resolution is increased by a factor of up to 2.6. We further analyzed the power spectrum density (PSD) curves of diffraction patterns obtained from labeled and control cells. The slope changes of the PSD curves indicate the noise level and information on labeling and diameter of the Au nanoclusters.

We chose the bacterium *Staphylococcus aureus* (*S. aureus*) as a model system to demonstrate the resolution enhancement. *S. aureus* occurs in grape-like clusters because the daughter cells do not fully separate and remain attached to one another during binary fission^26^. *S. aureus* can cause a wide range of illnesses, from minor skin infections to life-threatening diseases and is therefore important in clinical medicine worldwide^27^,^28^. A high-resolution structural understanding of these cells and of the relationship between the daughter cells is important to understand the pathogenicity of *S. aureus*. Control and gold-labeled *S. aureus* were imaged separately using the XFEL-CDI method shown in Fig. 1. This enhanced CDI method provides a feasible path for improving the resolution of biomaterial imaging and can reveal dynamic processes at high resolution.

## Results

### Labeling *S. aureus* with Au nanoclusters

Some nanoclusters that are considered biocompatible^29^ have various applications in biology due to their special physical, chemical and optical properties^30^,^31^,^32^,^33^, these applications include fluorescent labeling, drug delivery, heating, sensing and imaging. Acute gold (Au) nanocluster cytotoxicity has not been observed^29^. In our experiments, *S. aureus* were grown in Luria-Bertani broth at 37 °C. Control and labeled *S. aureus* were freshly prepared (Methods), and ultrathin sections of control and labeled *S. aureus* were then studied by cryogenic transmission electron microscopy (cryo-TEM) separately (Methods). Figure 2 shows the labeling of *S. aureus* using Au nanoclusters. In comparison to control *S. aureus*, some high-density black dots (indicated by red arrows) with a mean diameter of ~9.2 nm are found in the test sample (Fig. 2c). These black dots, which were confirmed to be Au nanoclusters using energy-dispersive spectroscopy (EDS), were found uniformly and randomly not only on the surface but also inside *S. aureus*. Each *S. aureus*–labeled cell contained ~5000 Au nanoclusters based on a mass spectrometric analysis, corresponding to approximately 10–20% mass fraction. Based on a cryo-TEM analysis, there were no obvious structural differences between the control and labeled *S. aureus* (such as cell wall or plasma membrane changes).

### Single-shot enhanced CDI experiment

**Figure 1:**
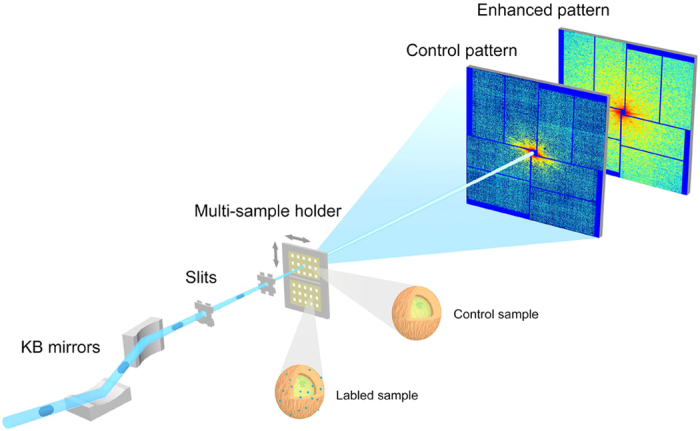
Schematic layout of the enhanced single-pulse coherent diffraction imaging experiment. In front of the sample chamber, a pair of Kirkpatrick-Baez mirrors was used to focus the X-ray pulse to ~2 × 2 μm^2^. Two sets of slits with beveled edges were positioned in front of the sample to remove the stray scattering. Two Si_3_N_4_ membranes in which control and gold-labeled *S. aureus* were deposited were mounted on a multi-sample holder. While scanning the membranes relative to the focused XFEL pulses, the control and enhanced diffraction patterns were recorded using an MPCCD downstream of the sample stage. All devices were under a vacuum of 10^−4^ Pa.

Enhanced CDI experiments on *S. aureus* were conducted using the SPring-8 Angstrom Compact Free-electron Laser (SACLA)^34^ and a multiple-application X-ray imaging chamber^35^ (Fig. 1). The XFEL pulses were focused to a spot size of ~2.0 μm × 2.0 μm. A photon energy of 5.5 keV, a pulse duration of ~10 fs, and a repetition rate of 10 Hz were used, and the typical pulse energy was ~100 μJ at the sample spot. Control and Au nanocluster-labeled *S. aureus* were deposited onto 50-nm-thick Si_3_N_4_ membranes for data collection (Supplementary Fig. 1). Diffraction patterns were recorded using a multi-port, charge-coupled device (MPCCD) detector^36^ located 2710 mm downstream of the sample stage (Methods). In our enhanced CDI experiment, a multi-sample holder with control and labeled *S. aureus* was mounted on the sample stage as shown in Fig. 1. While scanning the membranes relative to the focused XFEL pulses, the diffraction patterns from the control and Au nanocluster-labeled *S. aureus* specimens were successfully recorded by the MPCCD. The hit rate was only a few percent and was determined by the concentration of *S. aureus* on the membranes and the spot size of the beam. Although the hit rate was low, due to the fast imaging speed and high efficiency of XFEL, a number of diffraction patterns were recorded, including single-shot, multiple-shot and partial-shot patterns. By optimizing the concentration of the samples that were deposited on the membranes and using a more accurate micro-applicator, the hit rate could be improved significantly.

### Image reconstruction from the diffraction patterns

A number of diffraction patterns from both labeled and control samples were recorded. Due to the large missing central data and other factors, most of the diffraction patterns could not be reconstructed. Figure 3a,c show representative and reconstructable diffraction patterns of control and Au nanocluster-labeled *S. aureus* after data processing (Methods). The missing low angle data in Fig. 3a,c are due to the central hole of the MPCCD, which was designed to protect the detector from the direct XFEL pulse. By optimizing the center hole size, the partial first diffraction speckle of the control and labeled diffraction patterns was reserved. Reconstructions from the diffraction patterns (Fig. 3a,c) were performed using a hybrid input-output (HIO) phase retrieval algorithm combined^37^ with loose and tight supports (Methods). When missing data is restricted to the central diffraction speckle, reconstructions with better consistency are usually easier to obtain. When more data is missing, the patterns require special treatment to obtain reconstructions with better consistency^38^. However, even if the partial first speckle is lost, reconstruction remains possible, especially with an accurate, tight support. On the other hand, for bar-like samples, sample orientation affects the reconstruction. Given the same missing central data, it is easier to obtain accurate reconstructions for samples with vertical or horizontal orientations than for tilted samples. Figure 3b shows the image of a control *S. aureus* strain reconstructed from the diffraction pattern shown in Fig. 3a; in this figure, two tightly connected daughter cells can be recognized based on their profiles and inner density distribution. The pixel size of the reconstructed image is 16.5 nm. Because more data is missing in the Au nanocluster-labeled patterns, the reconstruction is more difficult for these samples. Compared with the other Au nanocluster-labeled patterns, the orientation of the pattern in Fig. 3c is close to horizontal, which indicates that the *S. aureus* strain is vertical. The approximate horizontal orientation was helpful for reconstruction because more useful data was preserved, and a more accurate initial rectangular support was obtained. Figure 3d presents a reconstruction from the corresponding diffraction pattern of Au nanocluster-labeled cells (Fig. 3c) (pixel size = 6.2 nm). The difference between two independent reconstructions (Supplementary Fig. 2) was calculated as ~2.15% based on Equation 1.


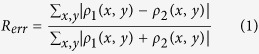


Here, *ρ*_1_ and *ρ*_2_ represent the two independent reconstruction. Line scans along the same position of *S. aureus* indicate excellent consistency between the two reconstructed images (Supplementary Fig. 2c). To further confirm our reconstructions, scanning electron microscope (SEM) imaging and scanning transmission X-ray microscope (STXM) imaging (Methods) of other *S. aureus* strains on the same membrane were performed (Fig. 3e,f, Supplementary Fig. 4). The STXM and SEM results of labeled *S. aureus* show the reconstruction results including a similar strain profile and inner density distribution (Fig. 3e,f). Another two pairs of STXM and SEM results (control and labeled *S. aureus*) also show the same results (Supplementary Fig. 4). The slight differences of the inner structure between the reconstructed results and the STXM images are due mainly to differences between cells.

### Resolution of the reconstructed images

To quantitatively analyze the resolution of the reconstructed images, we first calculated the power spectral density (PSD) curves of the control and Au-nanocluster-labeled diffraction patterns separately, as shown in Fig. 4a. The blue curve corresponds to the labeled pattern, and the black curve corresponds to the control pattern. The PSD curves reflect the achievable spatial frequency of the diffraction signals. The PSD curve (Fig. 4a, blue line) indicates that the diffraction signal of the labeled diffraction pattern extends to a spatial frequency of 81 μm^−1^. Compared with the PSD curve obtained from the diffraction pattern of the labeled samples, the control pattern signals achieve a spatial frequency of approximately only 45 μm^−1^. The PSD curves in Fig. 5e also show the similar results. Based on the PSD curves, the noise level of the diffraction pattern was estimated at approximately 0.1 photons per pixel. Because the signals extend to 81 μm^−1^ for the labeled pattern and to 45 μm^−1^ for the control pattern, the theoretical full-period resolution is expected to be 12.4 nm for labeled *S. aureus* and 22 nm for control *S. aureus*. However, the spatial frequency analysis just indicates the highest signal intensity, which reflects the theoretical achievable reconstruction resolution. Due to noise, missing data, and other factors, the resolution achieved is usually worse than the theoretical resolution that is calculated based on the highest angle signal measured. To further quantitatively estimate the achievable resolution, the phase retrieval transfer function (PRTF)^39^ curves of reconstructed images were calculated. However, the initial phase and missing data affect the reconstruction resolution and consistency. To remove random phase effects, 100 independent reconstructions of labeled and control *S. aureus* cells were averaged to calculate the PRTF curves. The blue and black curves shown in Fig. 4b represent labeled and control PRTF, respectively. Based on the criterion of PRTF = 1/e (black dashed line)^22^, the achievable full-period resolution is approximately 54 nm for labeled *S. aureus* and is 143.5 nm for control *S. aureus*.

### Radiation dose

For biological samples, such as proteins, biological assemblies, and cells, the achievable resolution in CDI is limited by the X-ray exposure dose, which affects the level of radiation damage. Femtosecond XFEL can overcome the problems associated with radiation damage by recording diffraction patterns before sample destruction^18^,^40^. In our experiment, the average radiation dose given to the samples in a single pulse was estimated at approximately 110 MGy, which far exceeds the radiation dose limit for biological structures^41^. However, in femtosecond single-pulse imaging, the available resolution is no longer limited by radiation damage, and higher resolution can be achieved since the data is collected before onset of the effects of radiation damage.

### Enhancement by labeling *S. aureus* with Au nanoclusters

Even using ultra-bright XFEL sources, the capture of high-resolution images of non-periodic biomaterials remains challenging because of the low scattering ability of these materials^22^,^24^. In previous studies, references or templates were used to increase diffraction signal intensity and to improve the resolution of the reconstructions of low-scattering samples^42^,^43^. By introducing references or templates, reconstruction becomes easier, and resolution is improved at least by a factor of 2, depending on the simulation results^44^,^45^,^46^. Nanocluster labeling is widely used for cells for intercellular imaging and tracking. Beyond labeling, intracellular biomineralization occurs widely in many types of microorganisms, including the biomineralization of Au nanoparticles in metallophilic bacteria and the biomineralization of high-purity magnetic minerals such as magnetite (Fe_3_O_4_) and greigite (Fe_3_S_4_) in magnetotactic bacteria^47^,^48^. The quantitative imaging of magnetosomes inside magnetotactic bacteria using synchrotron radiation based coherent diffraction imaging has been performed, yielding high-contrast images^49^. The use of intracellular high-atomic-number nanoclusters is a meaningful way to increase effective imaging resolution. Labeling with high-atomic-number nanoclusters provides an efficient approach to solve this challenge under available conditions by increasing the X-ray coherent scattering cross-section to enhance diffraction signals and to improve resolution. In these experiments, Au nanocluster-labeled *S. aureus* were used to demonstrate the enhancement of resolution by CDI using XFEL pulses. The diffraction patterns obtained for control and labeled *S. aureus* under the same conditions were recorded as shown in Figs 3a and 5a,b (control patterns) and Figs 3c and 5c,d (labeled patterns).

For a finite object illuminated by a coherent X-ray, the far-field diffracted waves fulfill the Born approximation. The form factor is the Fourier transform of the object intensity, as shown in Equation 2:


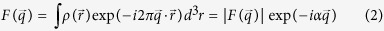


where 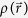 is the object electron density, 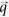 is the spatial frequency, and 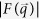 is the amplitude. The diffraction intensity recorded by the CCD detector element subtending the solid angle ΔΩ at diffraction angle *θ* is:


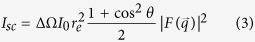


*I*_0_ is the incident X-ray intensity, and *r*_*e*_ is the classical radius of an electron. The scattering intensity at diffraction angle *θ* is proportional to the incident X-ray intensity and to the square of the form factor:


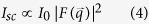


The incident X-ray intensity *I*_0_ is dependent on the XFEL single pulse intensity, which does not change significantly. Increasing the amplitude of the form factor 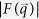 is an efficient way of increasing the diffraction signal intensity. Carbon, nitrogen, oxygen and hydrogen are the main elements comprising biomaterials. Figure 5f shows the form factors of these elements and of Au^50^,^51^. Carbon is the most frequent element in biomaterials; based on a comparison of the form factors of carbon and Au, the scattering ability of Au is approximately 170 times that of carbon. By labeling cells with high-atomic-number nanoclusters, the mean electron density 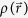 can be increased, thereby increasing the diffraction signal intensity *I*_*sc*_.

Comparison of the control and labeled diffraction patterns showed that the diffraction signals of the labeled *S. aureus* cells were much stronger than those of the control cells. The diffraction signals of the labeled *S. aureus* cells extended to the edge of the MPCCD. However, due to missing data and sample orientation, the diffraction patterns in Fig. 5 could not be reconstructed. To further compare the signal enhancement obtained by labeling, the PSD curves of the patterns obtained from both the control and labeled cells were calculated, as shown in Fig. 5e. Before analyzing the diffraction signal intensity, we eliminate the uncertain factors that could affect the signal intensity, such as exposure time, light source brightness and total electron density of the imaged samples. For single XFEL pulses, the fluctuation is less than 10 percent and the pulse duration is 10 femtosecond. The electron density is different in various samples. For instance, the sample size reflects the total electron density in some degree. In our experiment, the optical images of the control and labeled *S. aureus* shown in Supplementary Fig. 1 indicate that the size of the control and labeled *S. aureus* strains is similar. Besides, according to the autocorrelation results shown in Supplementary Fig. 5, the size of the control sample is even slightly larger than the labeled sample. The PSD curves shown in Figs 4a and 5e indicate that the diffraction signals of the labeled *S. aureus* cells were significantly stronger than those signals of the control cells. A quantitative intensity difference of these two diffraction patterns was calculated by dividing labeled PSD curve with the control, shown in Supplementary Fig. 6b. The mean signal intensity of the labeled one is about 2.4 times that of the control.

To further quantitate the signal enhancement, we calculated the coherent scattering cross-section of the control and the labeled *S. aureus* cells according to the bacterial model and the amount of labeled Au nanoclusters^51^,^52^. The coherent scattering cross-section of the control *S. aureus* cells was 0.35 cm^2^/g, whereas that of the labeled *S. aureus* cells comprising a mass fraction of 10% Au nanoclusters (~5000 particles per cell) was 1.05 cm^2^/g. The coherent scattering cross-section of the labeled *S. aureus* was therefore increased by a factor of 3.14.

**Figure 2:**
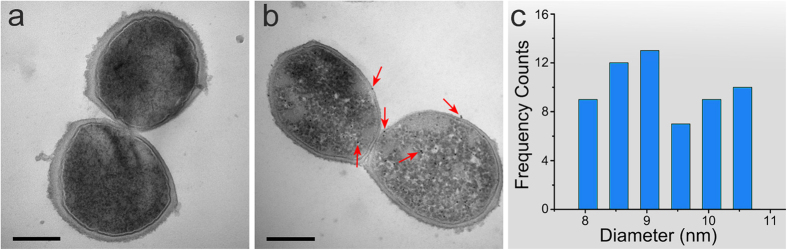
*S. aureus* cells with and without Au nanocluster labeling. (**a,b**) Cryo-TEM images of ultrathin sections of control and Au nanocluster-labeled *S. aureus* cells. In comparison to the control, the cryo-TEM image of the labeled *S. aureus* cells (**b**) shows that the Au nanoclusters (red arrows) were located both inside and on the surface of *S. aureus*. Scale bars, 300 nm. (**c**) The size of the Au nanoclusters ranged from 8 to 10.6 nm with a mean diameter of 9.2 nm.

Based on the PSD curves shown in Fig. 5e, the spatial cutoff frequency of the control patterns is approximately 43 μm^−1^, corresponding to ~11.6 nm of theoretical spatial half-period resolution, whereas the spatial cutoff frequency of the labeled patterns extends to the edge of the CCD, corresponding to ~6.2 nm of spatial half-period resolution. Beyond the cutoff frequency of the control patterns, the PSD curves contain noise of approximately 0.1 photons per pixel. In addition to the signal intensity of the PSD curves, the PSD curve slopes (which are calculated from the logarithmic scale of the diffraction intensity versus the logarithmic scale of the spatial frequency) are also different. According to theoretical calculation, a power law between the diffraction intensity *I*_*sc*_ and spatial frequency *f* takes the form *I*_*sc*_ ∝ *f*^*α* ^53,54. Here, the parameter *α* represents the slope of the PSD curves; this parameter takes a theoretical value of −4 for an idealized sample but in practice is measured in the range between −3 and −5^15^,^55^,^56^,^57^. At spatial frequencies of less than 20 μm^−1^, the slopes of the straight PSD curves are close to constant between −3.3 and −3.6, as shown in Figs 4a and 5e; this finding is consistent with theoretical results and indicates low signal-to-noise ratio at low spatial frequency. However, because the PSD curve slopes of the control patterns are more strongly affected by noise at high spatial frequency, the slopes tend to gradually change. The slopes of the labeled PSD curves above 20 μm^−1^ deviate from the reasonable range of about −4 to approximately −1 to −2. Because the control and labeled *S. aureus* differ by the presence of Au nanoclusters, the PSD curve intensity and slope differences between the diffraction patterns of the control and labeled cells are due to Au nanocluster labels. The PSD curves of the labeled patterns (Fig. 5e) exhibit one obvious peak at a spatial frequency of approximately 50 μm^−1^, which is characteristic ring of the diffraction pattern of Au nanoclusters. The characteristic ring in these patterns also indicates that the diameter of the Au nanoclusters is approximately 10 nm, a value that is consistent with the statistical result shown in Fig. 2c. The slopes of the PSD curves of the control and labeled cells were similar at low spatial frequency. Combined with the diffraction patterns obtained at low frequency (Fig. 5), the fringes along certain directions indicate that the samples adopted bar-like structures. Signal intensity is proportional to the square of the form factors, and Fig. 5f shows that the form factors decayed as spatial frequency increased. At low spatial frequency, the decay rates of Au and carbon are similar; a feature that is consistent with the similar PSD curve slopes obtained for the diffraction patterns of the control and labeled cells at low spatial frequency. Therefore, by labeling *S. aureus* with Au nanoclusters, the diffraction signals were increased significantly, thus extending the obtained spatial frequency by a factor of 2 (as determined based on the PSD curves). This result indicates that labeling with Au nanoclusters significantly enhanced the diffraction signals.

For the reference or template enhancement coherent diffraction imaging methods, interference between the sample and reference or template must be considered^44^,^45^. The recorded intensity is expected to be the following:


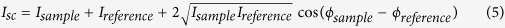


Because the signals from the reference or template are more intense than those from the samples, the interference term 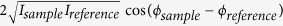 contributes to the enhancement of the signals of the sample. According to this model, the diffraction intensity of an Au nanocluster-labeled cell is expected to be the following:


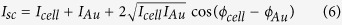


**Figure 3:**
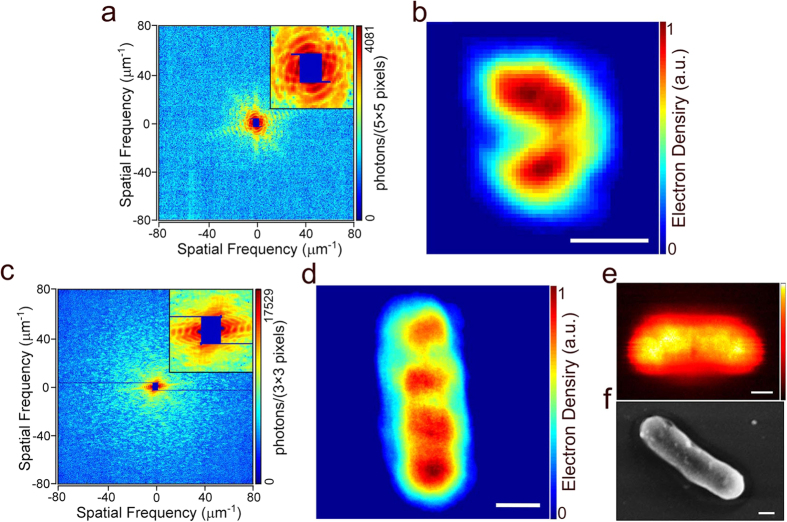
Reconstructions of *S. aureus* strains. (**a**) A representative diffraction pattern of control *S. aureus*. (**b**) A reconstructed image of an *S. aureus* cell from the diffraction pattern (**a**) shows two tightly connected daughter cells. (**c**) A representative diffraction pattern of labeled *S. aureus* cells, in which the diffracted signals extend to the edge of the MPCCD. (**d**) Four tightly connected daughter cells; a reconstruction result from (**c**) showing the diffraction pattern. (**e**) SEM image of another labeled *S. aureus* strain on the same membrane. (**f**) Scanning transmission X-ray microscopy image showing the morphology and density contrast of the labeled *S. aureus* cells. Scale bars, 300 nm.

**Figure 4:**
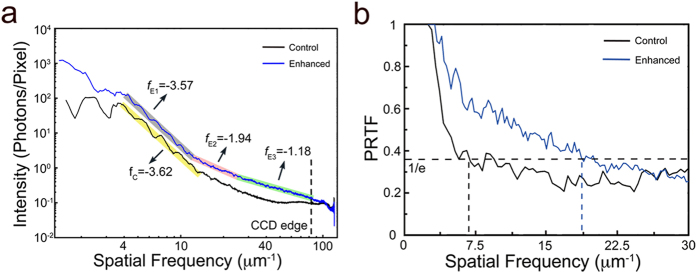
Reconstruction resolution and analysis. (**a**) Power spectral density (PSD) curves of the diffraction patterns shown in Fig. 3a,c. The black curve represents the control pattern, extending to a spatial frequency of 45 μm^−1^. The blue curve represents the labeled pattern and extends to the CCD edge. (**b**) Phase retrieval transfer function (PRTF) curves calculated from the reconstruction results. The black curve represents the control sample, and the blue curve represents the labeled sample. According to the criterion of PRTF = 1/e, the PRTF curves indicate a reconstruction resolution of 143.5 nm for the control reconstruction result and one of 54 nm for the labeled reconstruction result. The resolution was increased by at least a factor of 2.

**Figure 5:**
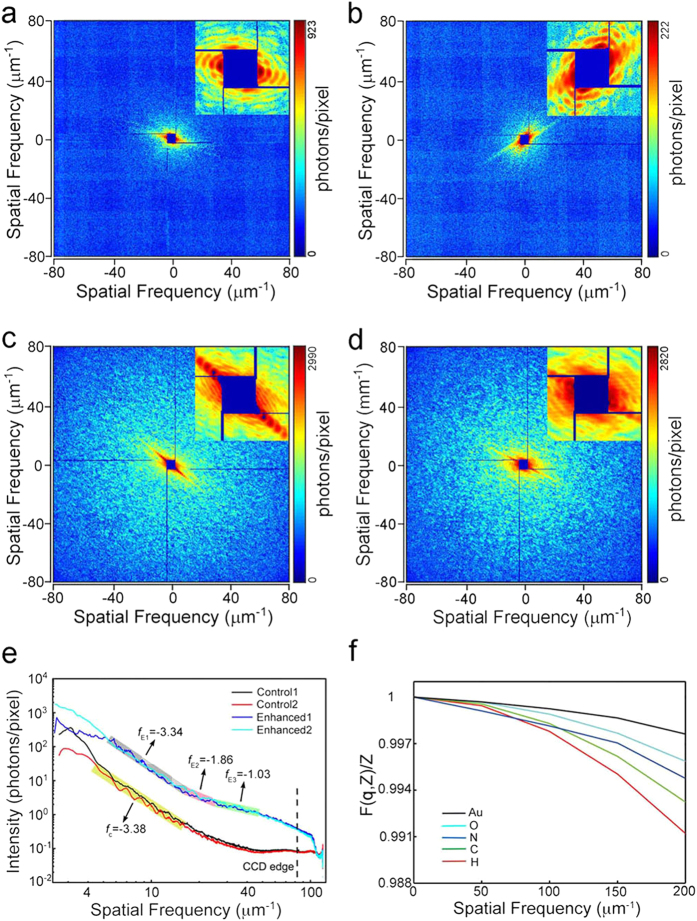
Diffraction enhancement of the single-pulse coherent diffraction imaging. (**a,b**) A pair of representative diffraction patterns of the control *S. aureus* cells. (**c,d**) Enhanced diffraction patterns of *S. aureus* cells that were labeled with Au nanoclusters, which have more valid signals and a higher signal to noise ratio than control patterns (**a,b**). (**e**) Power spectral density (PSD) of diffraction patterns (**a**–**d**). According to the PSD curves, the spatial cutoff frequencies for the control and labeled *S. aureus* cells are ~43 μm^−1^ and ~81 μm^−1^, respectively, indicating that the diffraction signals were improved by approximately 2 times. The slopes of the PSD curves represent signal decay rates. The signals of the labeled *S. aureus* cells decayed much more slowly than the control signals. (**f**) The form factors of carbon, nitrogen, oxygen, hydrogen and gold. Compared with the elements contained in biological samples, the Au form factor decays much more slowly, consistent with the PSD curve slopes.

The recorded intensity includes three parts: *I*_*cell*_, signals from the cell; *I*_*Au*_, signals from the Au nanoclusters; and 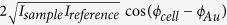, the interference term of the cell and Au nanoclusters. Based on the PSD curves, the decay rate of the signal from the labeled cells is much slower than that of the control, especially at high spatial frequency. The signals at high frequency are due primarily to the Au nanoclusters and the interference term of the Au nanoclusters and cells. The phase relationship of the interference term between the Au nanoclusters and the cells is also useful for the reconstruction. Based on a previous study of reference-induced resolution enhancement, the interference term is useful for increasing the signals and for reconstruction^45^. Further analysis indicated that the signal to noise ratio could be increased by overcoming instrument noise by introducing a reference^45^. Because more useful signals could be used for reconstruction, the achievable resolution should be improved. A comparison of the control and labeled reconstruction results shown in Fig. 3 indicates this conclusion. The resolution was increased at least by a factor of 2 both from the enhancement of the signals and the reconstruction results.

## Discussion

Although high-resolution imaging of biomaterials, such as whole cells, is affected by several factors, we demonstrated single-shot whole cell imaging using the enhanced coherent diffraction method and XFEL. By labeling *S. aureus* with Au nanoclusters, the diffraction signals were significantly enhanced. Using an HIO algorithm^37^ with loose and tight support, we successfully obtained a reconstructed image of *S. aureus* cells. Comparing the PSD curves for the diffraction patterns of the control and labeled cells, it is evident that the diffraction signals of the labeled cells were increased by at least a factor of 2. The reconstructions obtained from the diffraction patterns of the control and labeled cells indicate that the enhanced signals can also increase reconstruction resolution by at least a factor of 2. The slight difference between the theoretically calculated coherent scattering cross-section enhancement, signal enhancement and resolution enhancement may come from the experimental error, noise and missing central data. CDI is a quantitative imaging technique; the different colors in the reconstructed image represent different electron densities (Fig. 3b,d). Some bacterial structures can be studied based on the electron density distribution. *S. aureus* is a gram-positive bacterium, and few organelle types are present in the cell. The thick cell wall is a distinctive feature (Fig. 3b). Considering the thickness and composition of the sample, the high electron density areas shown in Fig. 3b,d are expected to be DNA-rich regions that contain mainly phosphorus. The labeled Au nanoclusters could not be distinguished in the reconstructed image due to the thickness of the cells and the small diameter of the nanoclusters.

For conventional CDI using synchrotron radiation, the achievable resolution of biomaterial imaging is limited by the potential for radiation damage. Thus, the use of longer exposure times does not always successfully improve spatial resolution. Our enhanced CDI experiments demonstrate that by labeling low-scattering *S. aureus* with Au nanoclusters, whole cells can be imaged using single pulses, and the resolution of the reconstructed images is increased by at least a factor of 2. Considering the issues of biocompatibility and biological universality^29^, labeling with an appropriate high-atomic-number element or nanoclusters of such atoms provides a promising way to enhance the resolution for cellular imaging experiments using XFELs. At present, because of limitations due to missing data and the low signal-to-noise ratio at high spatial frequency, the reconstructions obtained did not achieve the theoretical resolution based on the high angle diffraction signals detected. Using stronger XFEL pulses, a higher dynamic range and improved detector, the area of missing data could be restricted to much smaller regions, and better signal-to-noise ratio might be obtained; therefore, higher resolution images of labeled whole cells using single pulses is achievable.

## Methods

### Preparation of *Staphylococcus aureus*

*S. aureus* was grown in Luria-Bertani broth at 37 °C and collected in mid-exponential growth phase. The cells were centrifuged at 3600 rpm/min for 2 min at 4 °C to remove residual culture medium, and the *S. aureus* cells were then washed three times with saline solution (0.9% NaCl) and three times in deionized water. HAuCl_4_ (25 mmol/L) was added to the concentrated bacterial pellet, and NaOH solution (0.5 mmol/L) was introduced to adjust the pH to 7. The *S. aureus* solution was stirred at 200 rpm/min for 0.5 hours to obtain good dispersion. After incubating with gold chloride acid solution for 4 hours, Au nanoclusters were grown in *S. aureus*. The *S. aureus* dispersion was centrifuged at 3600 rpm/min for 2 min at 4 °C and then washed three times with saline solution (0.9% NaCl) and three times with deionized water to remove residual gold salts. Then bacterial cells were then fixed using 5% glutaraldehyde for 4 hours. The number of labeled Au nanoclusters in each *S. aureus* cell was determined to be ~5000 based on mass spectrometric analysis.

After fixation, the control and labeled *S. aureus* cells were suspended in deionized water and supported on 50-nm-thick Si_3_N_4_ membranes. Before the CDI experiments, an optical microscope was used to check the density of *S. aureus* on the membranes. One major advantage of using a membrane is that background subtraction is effective because of the clean and uniform background of the membranes.

### Enhanced Coherent Diffraction Imaging

Enhanced CDI was conducted at BL3 of the SPring-8 Angstrom Compact Free-Electron Laser (SACLA). During the experiments, membranes containing control and labeled *S. aureus* were separately mounted on the sample stage and exposed to single XFEL pulses (5.5 keV photon energy, 10 fs pulse duration and 10 Hz XFEL repetition rate). As the samples were rastered across the X-ray beam between the collection of images, an experimental repetition rate of approximately 1 Hz was obtained. To eliminate scattering from air, the experiment was performed under vacuum (10^−4^ Pa). In front of the sample chamber, a pair of Kirkpatrick-Baez (KB) mirrors was used to focus the X-ray beam to a spot size of ~2.0 × 2.0 μm^2^ at the sample position. Two sets of independent cross-slits were located in front of the sample stage to eliminate parasitic scattering from the KB mirror and other optical devices. At 2710 mm downstream of the sample, a multiport charge-coupled device (MPCCD) octal sensor (2048 × 2048 pixels; pixel size, 50 × 50 μm^2^) was used to record the diffraction pattern. To protect the MPCCD from the direct beam, the central aperture of the MPCCD was set at 3.5 × 3.5 mm^2^. After all devices were aligned, 2D scans of the Si_3_N_4_ membrane were performed using single XFEL pulses with a 1 Hz repetition rate (which was decayed using a pulse selector). The membranes containing the samples were destroyed after exposure to the XFEL pulses, leaving holes (Supplementary Fig. 1).

### Data analysis and reconstruction

In our measurements, we acquired a large quantity of single-pulse diffraction patterns, although the probability of hitting the samples was low for each X-ray pulse. The coherent diffraction patterns indicative of hitting *S. aureus* cells were selected by measuring the mean intensity in a central region of 200 × 200 pixels. By setting a threshold of mean intensity, diffraction patterns containing stronger signals were selected. Then we manually checked the diffraction patterns to distinguish the diffraction patterns of single *S. aureus* strains from diffraction patterns of multiple strains. In the optical images and SEM images of *S. aureus*, the profiles of the *S. aureus* strains were mostly bar-like. Some strains contained two contacting *S. aureus*, as shown in Fig. 3b. According to the bar-like strain profile, the single shot diffraction patterns should show fringes in certain directions. Based on this standard, we selected diffraction patterns of single *S. aureus* strains.

Background subtraction was performed using diffraction patterns from clean regions of the same membrane. After subtracting the backgrounds from the experimental diffraction patterns, high signal-to-noise ratio diffraction patterns of *S. aureus* were obtained. To further improve the signal-to-noise ratio and iterative speed, 3 × 3 pixel binning was performed for the labeled diffraction patterns, as shown in Fig. 3c; 5 × 5 pixel binning for control diffraction patterns was also used, as shown in Fig. 3a.

The reconstructions were performed using a hybrid input and output algorithm with a typical *β* of 0.8. Because the central are of missing data in the diffraction patterns was somewhat large, a relatively tight support was important for successful reconstruction. First, the autocorrelation functions of the diffraction patterns of the control and labeled cells were calculated to obtain the morphologies of the *S. aureus* strains and to determine the initial rectangular supports. After 10,000 iterations, a stable reconstruction result was achieved. The five reconstructions with the smallest Fourier-space errors^37^ were selected and averaged to calculate a relatively tight support. We aligned the relative positions of these five reconstructions using the center-of-mass alignment method. Setting a threshold of 0.1, a tight support was calculated from the averaged image. To eliminate the artificial effects by introducing the threshold, 5 pixels were extended from the tight support. Then, the tight support was used to perform another 10,000 iterations, and a more accurate reconstruction was achieved. Using the same procedure, we updated the tight support to obtain a more accurate tight support. For the updated tight support, a 0.1 threshold was used, and the support was extended by 3 pixels. Using the updated support, 500 reconstructions were performed with 10,000 iterations for each reconstruction. The 100 reconstructions with the smallest errors were selected as the final reconstruction result to calculate the PRTFs.

### Cryogenic Transmission Electron Microscopy (cryo-TEM) and Scanning Electron Microscopy (SEM)

The bacterial cells were fixed in osmium tetroxide for 90 min. The specimens were further dehydrated in a series of alcohol solutions and embedded and polymerized in araldite for 24 hours. The specimens were then cut into ultrathin sections and deposited on a copper grid. Finally, the specimens were treated with 2% uranyl acetate for 15 min and lead citrate for 5 min. Cryo-TEM (H-7650, HITACHI, Japan, 80 KV) and selected area energy-dispersive spectroscopy (SAEDS) were used to observe control and labeled *S. aureus* cells.

The control and labeled *S. aureus* cells on the membranes used for the XFEL experiment were observed using SEM (Hitachi S-4800, Japan, 5 KeV). The presence of Au in the bacteria after gold chloride acid treatment was verified using energy-dispersive spectroscopy (EDS).

### Scanning Transmission X-ray Microscopy

Imaging of *S. aureus* using scanning transmission X-ray microscopy was conducted at BL 08U of the Shanghai Synchrotron Radiation Facility (SSRF). A scanning transmission X-ray microscope was used to record images by monitoring the X-ray signal transmitted through the specimens. The beam size was focused to ~30 nm using a zone plate. An order-sorting aperture (OSA) was placed between the zone plate and specimen, and only first-order focus was selected. Membranes which control and labeled *S. aureus* cells were deposited on and used in a single-pulse coherent diffraction imaging experiment were mounted on a sample stage at the first-order focal spot. STXM images of the control and labeled *S. aureus* cells were acquired using a 15-nm scan step.

## Additional Information

**How to cite this article**: Fan, J. *et al*. Single-pulse enhanced coherent diffraction imaging of bacteria with an X-ray free-electron laser. *Sci. Rep.* **6**, 34008; doi: 10.1038/srep34008 (2016).

## Supplementary Material

Supplementary Information
