# Supplementary material for: Single-pulse enhanced coherent diffraction imaging of bacteria with an X-ray free-electron laser
Source: Sci Rep. 2016 Sep 23;6:34008. doi: 10.1038/srep34008 (PMC5034275; doi:10.1038/srep34008)
Supplement: Supplementary Information [file srep34008-s1.doc]

Supplementary Information

**Single-pulse enhanced coherent diffraction imaging of bacteria with an X-ray free-electron laser**

Jiadong Fan^1^, Zhibin Sun^1^, Yaling Wang^2^, Jaehyun Park^3^, Sunam Kim^3^, Marcus Gallagher-Jones^3,4^, Yoonhee Kim^5^, Changyong Song^6^, Shengkun Yao^1^, Jian Zhang^1^, Jianhua Zhang^1^, Xiulan Duan^1^, Kensuke Tono^7^, Makina Yabashi^3^, Tetsuya Ishikawa^3^, Chunhai Fan^8^, Yuliang Zhao^2^, Zhifang Chai^2^, Xueyun Gao^2*^, Thomas Earnest^8,9^ & Huaidong Jiang^1,10*^

^1^State Key Laboratory of Crystal Materials, Shandong University, Jinan 250100, China. ^2^Chinese Academy of Sciences Key Laboratory for Biomedical Effects of Nanomaterials and Nanosafety, Institute of High Energy Physics, Chinese Academy of Sciences, Beijing 100049, China. ^3^RIKEN SPring-8 Center, Kouto 1-1-1, Sayo, Hyogo 679-5148, Japan, ^4^Institute of Integrative Biology, University of Liverpool, Liverpool L69 7ZB, United Kingdom, ^5^School of Materials Science and Engineering, Gwangju Institute of Science and Technology, Gwangju 500-712, Korea, ^6^Department of Physics, POSTECH, Pohang 790-784, Korea, ^7^Japan Synchrotron Radiation Research Institute (JASRI/SPring-8), 1-1-1 Kouto, Sayo, Hyogo 679-5198, Japan, ^8^Shanghai Synchrotron Radiation Facility, Shanghai Institute of Applied Physics, Chinese Academy of Sciences, Shanghai 201800, China. ^9^iHuman Institute, Shanghai Tech University, Shanghai 201800 China. ^10^School of Physical Science and Technology, ShanghaiTech University, Shanghai 201210, China.

| Supplementary Figure 1 | Si_3_N_4_ membranes with *S. aureus* before and after XFEL shots |
| --- | --- |
| Supplementary Figure 2 | Consistency of two independent reconstructions |
| Supplementary Figure 3 | Consistency of experimental and calculated diffraction patterns |
| Supplementary Figure 4 | STXM and SEM images of control and labeled *S. aureus* |
| Supplementary Figure 5 | Autocorrelation functions of single pulse diffraction patterns |
| Supplementary Figure 6 | Comparison of diffraction intensities between the control and labeled samples. |

**Supplementary figures**


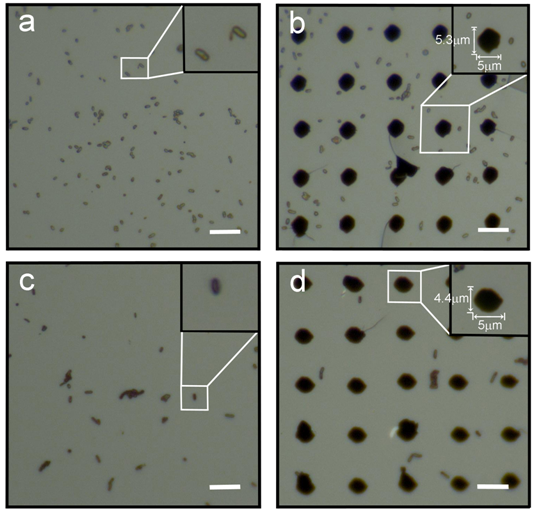


Supplementary Figure 1. Optical microscope images of Si_3_N_4_ membranes with *S. aureus* before and after XFEL shots. (a) Optical image of labeled *S. aureus* on a membrane. An inset shows an enlarged version of typical bar-like *S. aureus* strains which are similar to the reconstruction result. (b) After XFEL pulse shots, the Si_3_N_4_ membranes were broken due to ultrahigh X-ray intensity. The holes on the membrane represent rough beam profiles. (c,d) Optical microscope images of membranes with control *S. aureus* before and after XFEL shots. Scale bar, 10 μm.


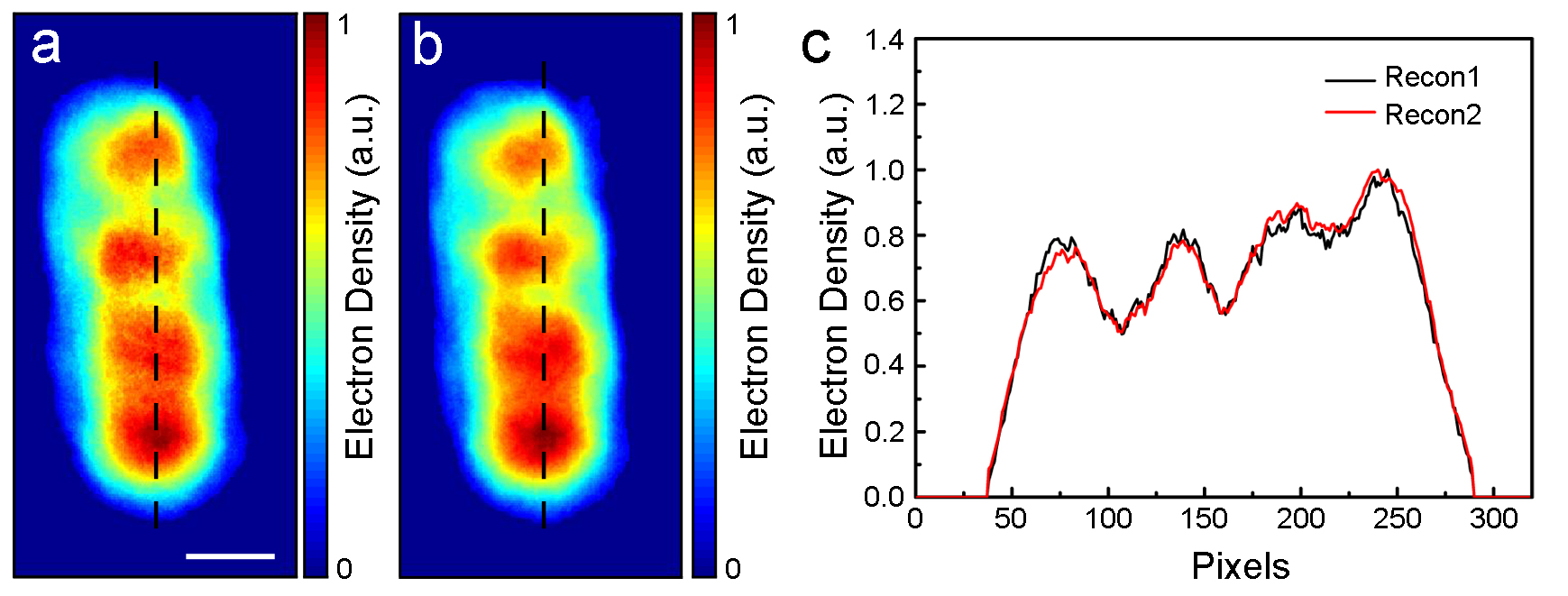


Supplementary Figure 2. Consistency of two independent reconstructions from the same diffraction pattern. (a,b) Two independent reconstructions with different initial phases. Both the profile and inner density distribution of *S. aureus* strain are similar. Based on the reconstructed images, a four-cell *S. aureus* strain can be recognized. Scale bar, 300 nm. (c) Two line scans along the same position of *S. aureus* strains shown in (a,b). The result indicates the independent reconstructions are in good agreement with each other. According to the equation $R_{err}=\frac{\sum_{x,y} \left|\rho_{1}(x,y)-\rho_{2}(x,y)\right|}{\sum_{x,y} \left|\rho_{1}(x,y)+\rho_{2}(x,y)\right|}$, the difference of the two reconstructed images is 2.15%.


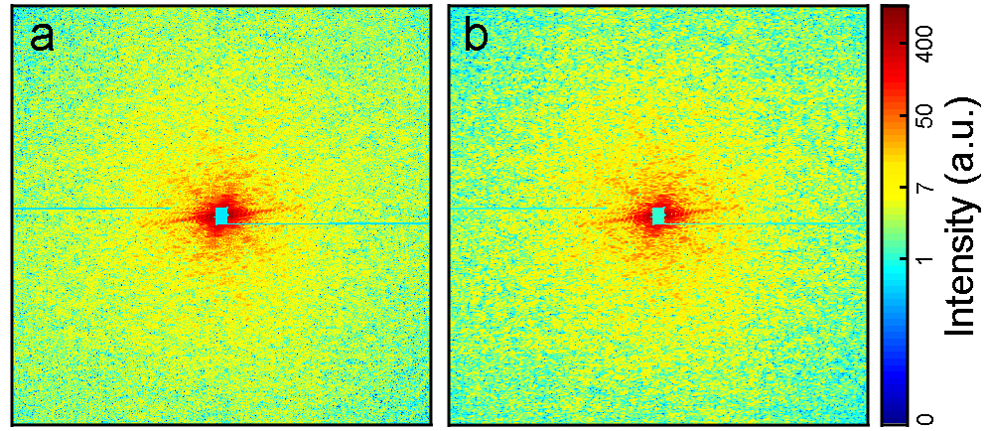


Supplementary Figure 3. Consistency of experimental and calculated diffraction patterns. (a,b) Measured and calculated diffraction patterns of *S. aureus*. Diffraction speckles and intensities of the calculated pattern are in good agreement with those of the measured diffraction pattern at both low and high frequency. The slight difference of the two patterns is due to noise in the experiment.


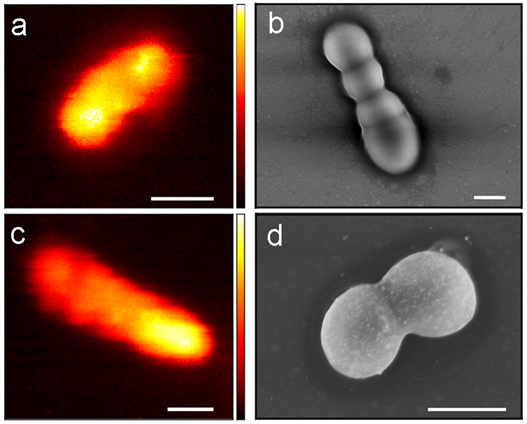


Supplementary Figure 4. Scanning transmission X-ray microscope (STXM) and scanning electron microscope (SEM) images of *S. aureus*. (a,b) STXM and SEM images of control *S. aureus* on the same membranes used for XFEL experiment. (c,d) STXM and SEM images of labeled *S. aureus* with Au nanoclusters. The STXM and SEM images indicate the strains are composed of about 2 to 5 *S. aureus* connected tightly one after another, which is consistent with the reconstructions. No appreciable difference was observed between the STXM images of control and labeled *S. aureus*, because STXM only provides absorption contrast images and the resolution is too low (about 30 nm) to see the Au nanoclusters. Scale bar, 500 nm.


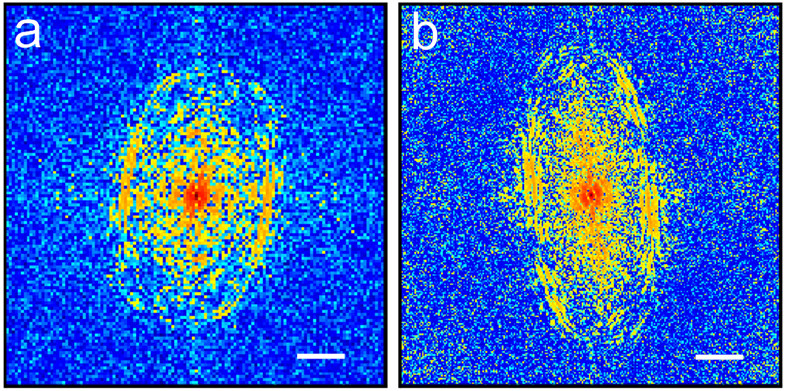


Supplementary Figure 5. Autocorrelation functions of single pulse diffraction patterns. (a) Autocorrelation function calculated from the diffraction pattern of control *S. aureus* shown in Figure 5a. (b) Autocorrelation function calculated from the diffraction pattern of labeled *S. aureus* shown in Figure 3c. A high pass filter was used to reduce the sharp cross effect because of the rectangular missing data in the central area. The autocorrelation functions reflect the size and shape of the *S. aureus* with symmetry of 180^o^ rotation but twice diameter in each direction compared with the actual *S. aureus* strains. By estimating the size of the samples based on the autocorrelation functions, the control is 1.14 times as large as the labeled. The size and shape of the autocorrelation function (b) are indicative of X-ray pulse hitting on a single *S. aureus* stain, which is in good agreement with the reconstruction result. Scale bar, 500 nm.


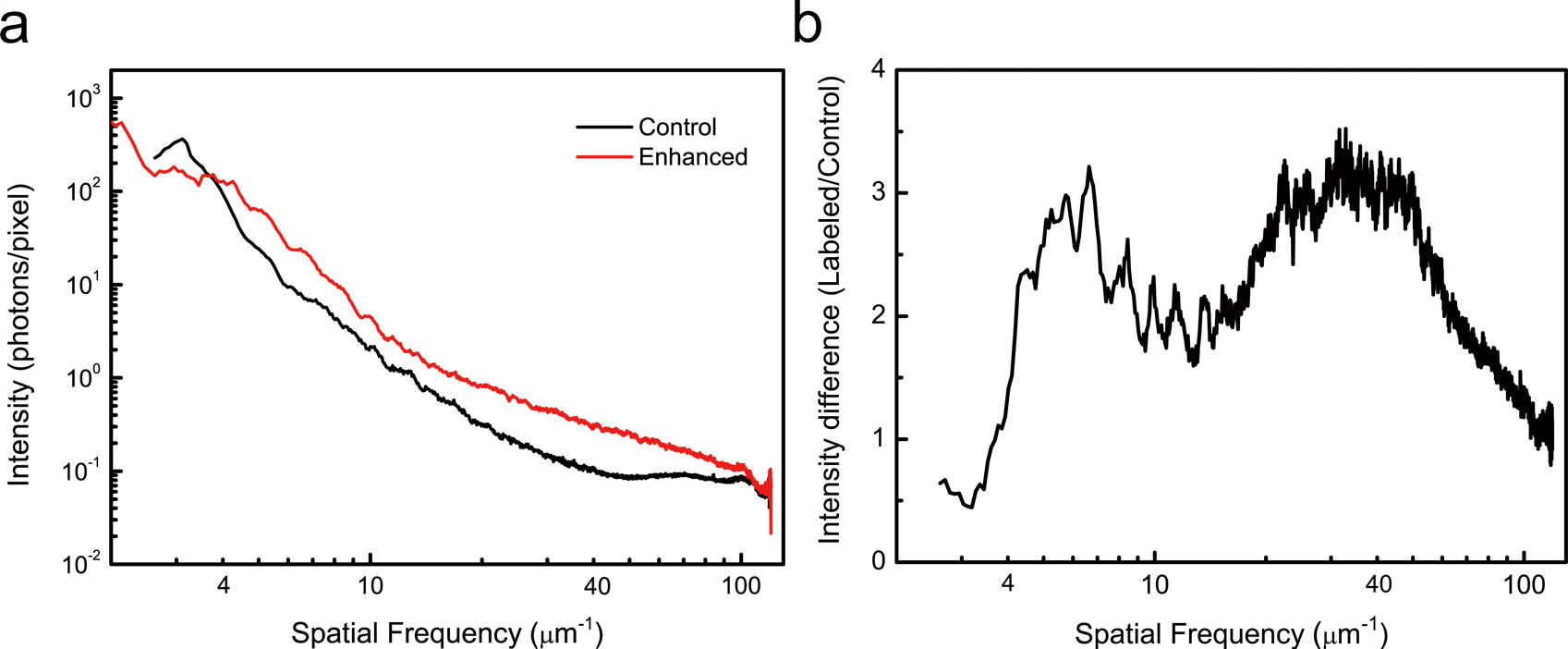


Supplementary Figure 6. Comparison of the diffraction intensities between the control and labeled samples. (a) The PSD curves of two typical control and labeled diffraction patterns shown in Figure 3c and Figure 5a. The red PSD curve is calculated from the labeled sample and the black one from the control sample. The size of these two samples was analyzed by using the autocorrelation function in supplementary Figure 5: the size of the control is about 1.14 times that of the labeled. However, the PSD curves show that the diffraction intensity of the labeled sample is obviously stronger than the control one at the same spatial frequency. The intensity fluctuations at low spatial frequencies are due to missing data. In order to show the intensity enhancement visually, an intensity difference curve was calculated by dividing the labeled PSD curve by the control PSD curve as shown in (b). This curve shows that the intensity of labeled diffraction pattern is about 2 times as strong as the control one. The peak at a spatial frequency of around 40 μm^-1^ is related to the enhancement due to the gold nanoclusters labeling.
